# Interventions for treating displaced intracapsular femoral neck fractures in the elderly: a Bayesian network meta-analysis of randomized controlled trials

**DOI:** 10.1038/s41598-017-13377-1

**Published:** 2017-10-12

**Authors:** Bin-Fei Zhang, Peng-Fei Wang, Hai Huang, Yu-Xuan Cong, Hu Wang, Yan Zhuang

**Affiliations:** Department of Orthopedic trauma, Honghui Hospital, Xi’an Jiaotong University, College of Medicine, Beilin District, Xi’an, Shaanxi Province China

## Abstract

Displaced intracapsular femoral neck (AO type 31 B2/3) fractures have various treatments, including internal fixation (IF), unipolar uncemented hemiarthroplasty (HA), bipolar uncemented HA, unipolar cemented HA, bipolar cemented HA, uncemented total hip replacement (THR), and cemented THR. Systematic literature retrieval was performed from the databases to compare them in a network meta-analysis. Forty studies (85 arms) containing 6141 patients were included. Overall, our network meta-analysis rank the orders of 7 procedures in reoperation, mortality, dislocation and infection, which indicates that IF may provide the highest reoperation incidence, unipolar cemented HA may provide the lowest reoperation incidence; uncemented THR contributes the highest dislocation incidence; and bipolar uncemented HA provides the lowest infection incidence. No differences in mortality were observed among the treatments. This conclusion is indirect; higher-quality direct comparisons are required.

## Introduction

Femoral neck fractures are among the most common orthopedic injuries in the elderly. In 1990, the estimated number of femoral neck fractures was 1.66 million worldwide, per year. Further, the incidence is increasing, with the number of femoral neck fractures projected to reach up to 6.26 million by the year 2050^1^. Compared with other fractures, femoral neck fractures exhibit specific consequences in blood supply, displacement and shear forces, often resulting in fracture nonunion^2^ and femoral head necrosis^3^. Even though the treatment history of femoral neck fracture is over 400 years old and has made considerable progress, numerous problems remain to be resolved because of above characteristics of the injury. Thus far, treatments for displaced intracapsular femoral neck fracture have included closed or open reduction and internal fixation (IF), hemiarthroplasty (HA), and total hip replacement (THR). Notably, the indications for particular treatment modalities are very heterogeneous among orthopaedic surgeons, although establishing algorithms and hospital care pathways have recently been focused on^4^. Great attention should be paid to treatment these fractures.

Numerous published randomized controlled trials (RCTs) concerning this topic have compared the efficacy of treatment among IF, THR, and HA^5–14^. In addition, many meta-analyses and systematic reviews have assessed comparisons of IF versus HA^15^, IF versus THR^16^, cemented HA versus uncemented HA^17^, unipolar HA versus bipolar HA^18^, and THR versus HA^19^. When considering uncemented and cemented HA, Parker *et al*. reported that unipolar uncemented HA offers less reoperation, similar mortality and function compared with IF^20^, while Hedbeck *et al*. demonstrated that unipolar cemented HA has less reoperation, similar complications and mortality, better health-related quality of life, compared with IF^11^. Deangelis *et al*. reported that the use of cemented or uncemented femoral components was associated with similar functional outcomes during 1 year^12^. When considering unipolar or bipolar, Hedbeck *et al*. described that unipolar and bipolar HA appeared to produce equivalent clinical outcomes after 1 year^21^. However, in contrast, bipolar HA resulted in better health-related quality of life beyond the first 2 years following surgery, compared to unipolar HA, in a study performed by Inngul *et al*.^10^. When considering uncemented or cemented THR, no direct evidence exists to evaluate relative performances. RCTs comparing uncemented and cemented THR have focused on whether either of these treatments provide better outcomes than IF in hip function^14,22^.

Therefore, all the 7 procedures of a direct or indirect comparison under displaced intracapsular femoral neck fractures lacked. Thus, to comprehensively evaluate the efficacy and complications of the 7 surgical procedures, we performed a Bayesian network meta-analysis via a global search of published RCTs on this topic, providing evidence for clinical decision-making.

## Methods

### Inclusion Criteria

(1) Trials: RCTs. (2) Participants: Elderly patients (≥65 years) suffering from displaced intracapsular femoral neck fractures. (3) Interventions: Interventions studied in the meta-analysis were IF, unipolar uncemented HA, bipolar uncemented HA, unipolar cemented HA, bipolar cemented HA, uncemented THR, and cemented THR. (4) Outcomes: reoperation, mortality, dislocation, infection. Specifically, the definition of reoperation was taken to be secondary surgery caused by any reason. Infection included both superficial and deep wound infection, but not respiratory or urinary tract or other systemic infection.

Exclusion criteria were as follows: patients being unfit for arthroplasty or IF, previous hip pathology (osteoarthritis, malignant disease, infectious disease), and the age was <65 year old.

### Literature Search

We searched PubMed (1966–2015.8), EMBASE (1974–2015.8), and the Cochrane library (Issue 8 of 12, August 2015) using a searching strategy that combined MeSH/Emtree terms and free text words: “Femoral Neck Fractures,” Femoral Neck Fractures, “Randomized Controlled Trial,” randomized controlled trial*, single-blind method, single blind*, double-blind method, double blind*, triple blind*, random allocation, random allocation*, randomly allocation*. Retrieval dates were from the time of database creation to 31 August 2015. The language should be English. The search strategy for Medline (Pubmed) was in Supplementary Table S1.

### Data Extraction and Quality Evaluation

Two investigators (Peng-Fei Wang and Hai Huang) were responsible for independently reading all titles, abstracts, and full texts using the following steps: (1) examining titles and abstracts to remove obviously irrelevant studies, (2) retrieving the full texts of potentially relevant trials, (3) examining the full texts for compliance with eligibility criteria, and (4) making final decisions on study inclusion and proceeding to data collection. From the studies included, the investigators extracted baseline information on subjects (e.g., treatment strategy, approach, and outcomes) and detailed methods used in the study design (e.g., publication year, study settings, designs, method of randomization, allocation concealment, and blinding). Disagreements were resolved via discussion with a third investigator (Hu Wang). When continuous variables were described as median, these values were translated into mean or aggregated depending on researcher recommendation^23^.

Each study was independently assessed for its methodological quality by the previous investigator. The criteria for methodological quality were based on those described in the Cochrane Reviewers’ Handbook 5.1.0^24^, including selection bias, performance bias, detection bias, attrition bias, reporting bias domains, random sequence generation, allocation concealment, blinding, incomplete outcome data, selective reporting, and other biases.

### Statistical Methods

The analysis was primarily based on Multiple Treatments Meta-analysis, as described by Salanti *et al*.^25^. We used the Bayesian method based on the Chaimani model for binary variables (utilizing random effects models). Statistical analysis of these variables was based on binomial likelihoods, with vague priors for the trial baselines, basic parameters (normal distribution with mean 0 and standard deviation 0.0001), and random effects standard deviation (uniformly distributed i in the interval 0 to 2). We used the Bayesian method based on the random effects of the Dias model for continuous variables. Statistical analysis was based on deviance contribution, with vague priors for the trial baselines, basic parameters (normal distribution with mean 0 and standard deviation 0.0001), and random effects standard deviation (uniformly distributed i in the interval 0 to 5). Comparative effectiveness of the treatments was expressed as the odds ratio (OR) or weighted mean difference (MD), with 95% credibility intervals (CrIs). The CrI is the Bayesian analog to confidence intervals used in traditional frequentist statistical approaches. We considered a result “significant” if the CrI did not include OR = 1. We also ranked meaningfully different procedures in terms of their likelihood of leading to the best results for each outcome. The meta-analysis was performed using Winbugs version 1.4.3 (Imperial College and MRC, UK). Further, we checked the consistency in closed loops using inconsistency factors and calculated the contribution of direct estimates effect of each pair in the entire network with STATA version 12.0 (STATA Corporation, College Station, TX, USA), under the traditional frequentist statistical approaches.

## Results

### Process for Selecting Trials

**Figure 1:**
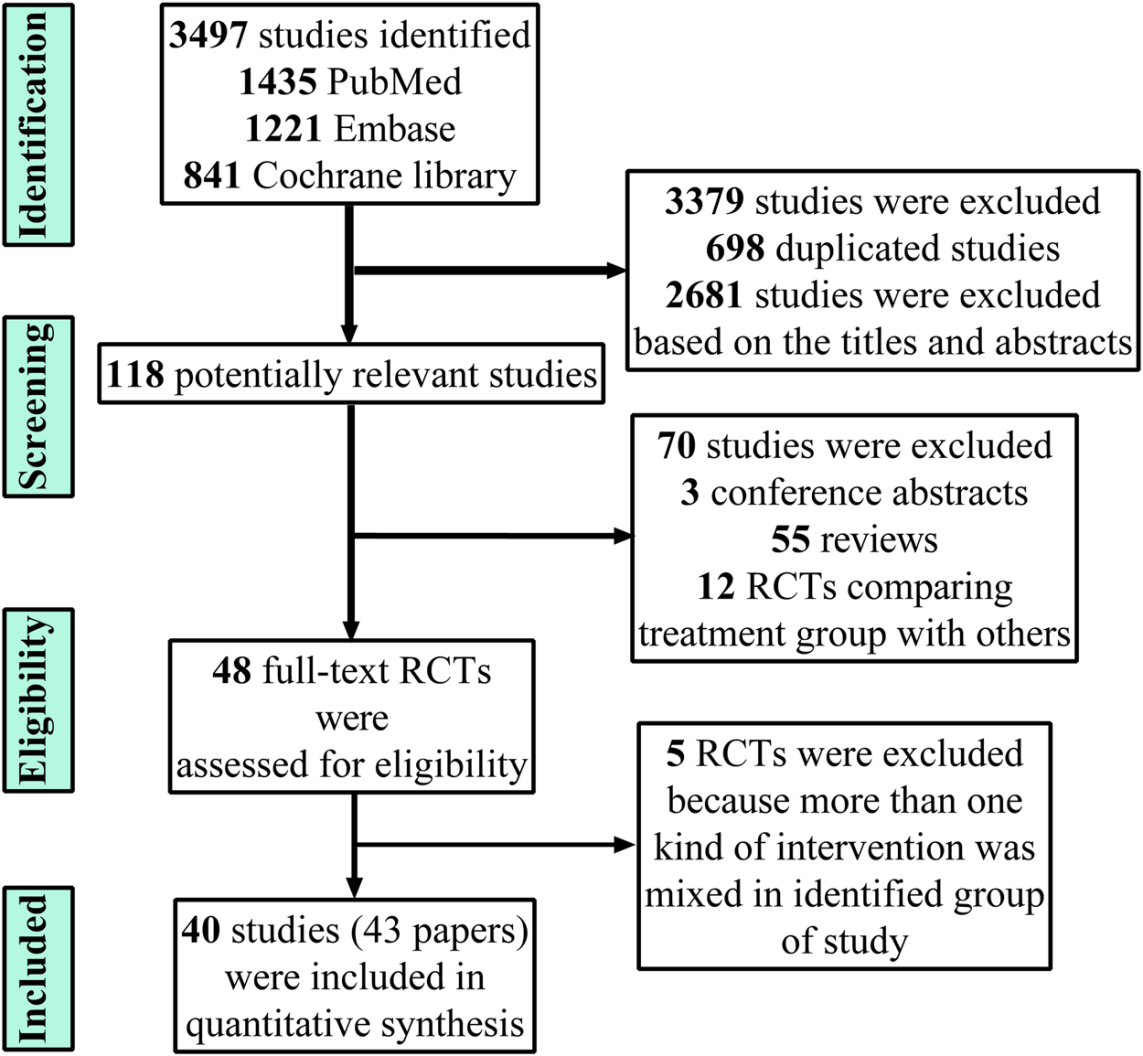
Flowchart of studies included in the meta-analysis.

In total, 3497 potentially relevant studies were identified and screened for retrieval (Fig. 1). From this initial study group, 698 studies were excluded because of duplications, and 2681 studies were excluded after reading their titles and abstracts. Among the remaining 118 studies, there were 3 conference abstracts, 55 reviews, and 12 RCTs comparing the treatment with others. Subsequently, 48 RCTs were assessed for eligibility. After reading, 5 RCTs were excluded due to more than 1 type of intervention being implemented in the study group. Finally, 40 studies (85 arms)^5–14,20–22,26–55^ were included in this meta-analysis.

### Characteristics of Included Trials and Quality Evaluation

The main characteristics of the included trials are listed in Table 1. The number of patients participating in studied RCTs varied from 20 to 455. A total of 6141 patients with femoral neck fractures were included in the meta-analysis; of these, the treatment breakdown included 1432, 1194, 821, 1511, 252, 229, and 702 in the IF, unipolar cemented HA, unipolar uncemented HA, bipolar cemented HA, bipolar uncemented HA, uncemented THR, and cemented THR groups, respectively. Five studies^32,36,43,50,51^ contained 3 arms; however, the remainder were 2-arm studies. In the IF group, cancellous lag screws or sliding hip screw (with or without antirotational screws) were used. In the other 6 groups, most patients underwent a modified Hardinge or a posterior approach. The follow-up period varied from 6 to 204 months. Eighteen studies did not report the number lost during follow-up^6,11,22,27,32–37,41–44,50–53^.

**Table 1:** Main characteristics of the trials included in the meta-analysis.

Study	Comparison			No. of patients	Age(years)	Female	Intervention or approach	Follow-up	Lost to follow-up
Blomfeldt 2005^54^; Tidermark 2003^29^	Uncemented-THR	IF		102(49/53)	79.2/81.4	40/42	Modified Hardinge approach TH R/Two cannulated screws	48 m	5
Blomfeldt 2007^55^	Bipolar-cemented-HA	Cemented-THR		120(60/60)	80.7/80.5	54/47	Modified Hardinge approach/Modified Hardinge approach	12 m	0
Calder 1996^53^	Unipolar-cemented-HA	Bipolar-cemented-HA		250(132/118)	85/85	114/101	Hardinge direct lateral approach/Hardinge direct lateral approach	12 m	NR
Cao 2014^14^	Uncemented-THR	IF		285(157/128)	75.9/76.8	84/69	Posterior approach/Three hollow compression screws	60 m	9
Chammout 2012^13^	Cemented-THR	IF		100(43/57)	78/79	38/41	Posterolateral approach/Two cannulated screws	204 m	23
Cornell 1998^52^	Unipolar-cemented-HA	Bipolar-cemented-HA		48(15/33)	77.6/78.0	11 + 25	Posterior approach/Posterior approach	6 m	NR
Davison 2001^51^	Unipolar-cemented-HA	Bipolar-cemented-HA	IF	280(90/97/93)	76/75/73	71/72/70	Lateral (Hardinge) approach/Lateral (Hardinge) approach/Lag screws	60 m	NR
DeAngelis 2012^12^	Unipolar-cemented-HA	Unipolar-uncemented-HA		130(66/64)	81.8/82.8	52/48	Modified Hardinge approach/Modified Hardinge approach	12 m	5
Dorr 1986^50^	Bipolar-cemented-HA	Bipolar-uncemented-HA	Cemented-THR	89(37/13/39)	72/66/69	26/9/23	Posterior approach/Posterior approach/Posterior approach	24 m	NR
Dortmont 2000^27^	Unipolar-cemented-HA	IF		60(29/31)	84/84	22/30	Anterior approach/Three cannulated screws	16.5 m	NR
Emery 1991^49^	Bipolar-cemented-HA	Bipolar-uncemented-HA		53(27/26)	78/79.6	24/22	NR	18 m	0
Figved 2009^48^; Langslet 2014^7^	Bipolar-cemented-HA	Bipolar-uncemented-HA		220(112/108)	83.4/83.0	87/80	Posterior approach/Posterior approach	24 m	0
Frihagen 2007^47^	Bipolar-cemented-HA	IF		222(110/112)	82.5/83.2	78/87	Lateral approach/Two parallel cannulated screws	24 m	17
Hedbeck 2011^21^	Unipolar-cemented-HA	Bipolar-cemented-HA		120(60/60)	87.4/85.5	49/42	Modified Hardinge approach/Modified Hardinge approach	12 m	1
Hedbeck 2011^46^	Bipolar-cemented-HA	Cemented-THR		120(60/60)	80.7/80.5	54/47	Modified Hardinge approach/Modified Hardinge approach	48 m	6
Hedbeck 2013^11^	Unipolar-cemented-HA	IF		60(30/30)	85.2/83.8	24/25	Anterolateral approach/Two cannulated screws	24 m	NR
Inngul 2013^10^	Unipolar-cemented-HA	Bipolar-cemented-HA		120(60/60)	87.4/85.5	49/42	Modified Hardinge approach/Modified Hardinge approach	48 m	61
Jeffcote 2010^45^	Unipolar-cemented-HA	Bipolar-cemented-HA		51(27/24)	81.4/80.1	21/18	Hardinge (antero-lateral) approach/Hardinge (antero-lateral) approach	24 m	10
Johansson 2000^9^	Cemented-THR	IF		100(50/50)	84/84	40/34	Dorsolateral approach/Two parallel and percutaneously-inserted screws	24 m	NR
Johansson 2014^22^	Cemented-THR	IF		146(68/78)	83.8/83.7	NR	Posterolateral approach/Two parallel and percutaneously inserted screws	180 m	0
Jonsson 1996^44^	Uncemented-THR	IF		47(23/24)	79/80	18/18	NR	24 m	NR
Kanto 2014^8^	Unipolar-cemented-HA	Bipolar-cemented-HA		175(88/87)	83.9/81.7	72/72	Posterior approach/Posterior approach	60 m	2
Keating 2006^43^	IF	Bipolar-cemented-HA	Cemented-THR	207(69/69/69)	74.3/75.0/75.2	51/54/52	Cannulated hip screws or a sliding hip screw/Lateral or posterior approach/Lateral or posterior approach	24 m	NR
Malhotra 1995^42^	Unipolar-uncemented-HA	Bipolar-uncemented-HA		68(36/32)	68/65	16/14	Moore’s posterior approach/Moore’s posterior approach	26 m	NR
Neander 1997^41^	Cemented-THR	IF		20(9/11)	77/71	6//5	Posterior approach/NR	18 m	NR
Øydna Støen 2014^5^	Bipolar-cemented-HA	IF		222 (110/112)	82/82	81/84	Lateral approach/Two parallel cannulated screws	72 m	2
Parker 2002^20^; Parker 2010^39^	Unipolar-uncemented-HA	IF		455(229/226)	82.4/82.2	183/181	Anterolateral surgical approach/Three parallel cannulated screws	36 m	0
Parker 2010^40^	Unipolar-cemented-HA	Unipolar-uncemented-HA		400(200/200)	83/83	161/147	Anterolateral approach/Anterolateral approach	6 m	3
Puolakka 2001^38^	Unipolar-cemented-HA	IF		32(15/17)	82/81	14/13	Posterolateral approach/Screws	24 m	0
Raia 2003^37^	Unipolar-cemented-HA	Bipolar-cemented-HA		115(60/55)	81.8/82.4	41/42	Posterolateral approach/Posterolateral approach	12 m	NR
Ravikumar 2000^36^	IF	Unipolar-uncemented-HA	Cemented-THR	271(91/91/89)	79.73/82.06/81.03	82/82/80	Screw/Posterolateral approach/Posterolateral approach	156 m	NR
Rödén 2003^35^	Bipolar-cemented-HA	IF		100(47/53)	81/81	37/34	Lateral approach/Bahr screws	120 m	NR
Santini 2005^34^	Bipolar-cemented-HA	Bipolar-uncemented-HA		106(53/53)	82.09/79.68	40/42	Lateral approach/Lateral approach	12 m	NR
Sikorski 1981^33^	Unipolar-cemented-HA	IF		190(114/76)	80.37/80.37	86/60	McKee anterolateral approach/Garden screws	24 m	NR
Skinner 1989^32^	IF	Unipolar-uncemented-HA	Cemented-THR	300(100/100/100)	80.9/80.9/80.9	90/90/90	Compression screw plate/Posterolateral approach/Posterolateral approach	12 m	NR
Somashekar 2013^6^	Unipolar-uncemented-HA	Bipolar-uncemented-HA		41(21/20)	75.57/67.35	10//17	Southern approach/Southern approach	12 m	NR
Stoffel 2013^31^	Unipolar-cemented-HA	Bipolar-cemented-HA		261(128/133)	81.9/82.9	NR	Hardinge lateral approach/Hardinge lateral approach	12 m	10
Taylor 2012^30^	Unipolar-cemented-HA	Unipolar-uncemented-HA		160(80/80)	85.3/85.1	57/53	Modified Hardinge approach/Modified Hardinge approach	24 m	45
van den Bekerom 2010^28^	Bipolar-cemented-HA	Cemented-THR		252(137/115)	80.3/82.1	115/90	Anterolateral, straight lateral or posterolateral	60 m	0
van Vugt 1993^26^	Bipolar-cemented-HA	IF		43(22/21)	76.0/75.3	14///11	Anterolateral approach/DHS	36 m	7

Most of the 40 studies reported random sequence generation from computerized randomization or random numbers (Table 2), there were 24 low risk, 1 high risk, and 15 unclear in random sequence generation. Allocation concealment was detailed in 23 studies, using the sealed-envelope technique, there were 23 low risk, and 17 unclear in allocation concealment. In blinding, there were 15 low risk, 16 high risk, and 9 unclear. In incomplete outcome data and selective reporting, all of studies were unclear.

**Table 2:** Quality evaluation of included trials.

Study	Random sequence generation	Allocation concealment	Blinding	Incomplete outcome data	Selective reporting	Other biases
Blomfeldt 2005^54^; Tidermark 2003^29^	Unclear	Low risk	High risk	Low risk	Unclear	Unclear
Blomfeldt 2007^55^	Unclear	Low risk	High risk	Low risk	Unclear	Unclear
Calder 1996^53^	Low risk	Unclear	High risk	Low risk	Unclear	Unclear
Cao 2014^14^	Low risk	Low risk	Low risk	High risk	Unclear	Unclear
Chammout 2012^13^	Low risk	Low risk	High risk	Low risk	Unclear	Unclear
Cornell 1998^52^	Low risk	Low risk	Low risk	High risk	Unclear	Unclear
Davison 2001^51^	Low risk	Unclear	Low risk	Low risk	Unclear	Unclear
DeAngelis 2012^12^	Low risk	Unclear	Low risk	Low risk	Unclear	Unclear
Dorr 1986^50^	Unclear	Unclear	High risk	Low risk	Unclear	Unclear
Dortmont 2000^27^	Unclear	Unclear	High risk	Low risk	Unclear	Unclear
Emery 1991^49^	High risk	Low risk	High risk	Low risk	Unclear	Unclear
Figved 2009^48^; Langslet 2014^7^	Low risk	Low risk	Low risk	Low risk	Unclear	Unclear
Frihagen 2007^47^	Low risk	Low risk	Low risk	Low risk	Unclear	Unclear
Hedbeck 2011^21^	Unclear	Low risk	High risk	Low risk	Unclear	Unclear
Hedbeck 2011^46^	Low risk	Low risk	Low risk	High risk	Unclear	Unclear
Hedbeck 2013^11^	Low risk	Low risk	Low risk	High risk	Unclear	Unclear
Inngul 2013^10^	Unclear	Low risk	High risk	Low risk	Unclear	Unclear
Jeffcote 2010^45^	Low risk	Unclear	High risk	Low risk	Unclear	Unclear
Johansson 2000^9^	Low risk	Low risk	Unclear	Low risk	Unclear	Unclear
Johansson 2014^22^	Low risk	Low risk	Unclear	Low risk	Unclear	Unclear
Jonsson 1996^44^	Low risk	Low risk	Unclear	Low risk	Unclear	Unclear
Kanto 2014^8^	Low risk	Low risk	Low risk	Low risk	Unclear	Unclear
Keating 2006^43^	Low risk	Low risk	Unclear	Low risk	Unclear	Unclear
Malhotra 1995^42^	Unclear	Unclear	Unclear	Low risk	Unclear	Unclear
Neander 1997^41^	Unclear	Unclear	High risk	Low risk	Unclear	Unclear
Øydna Støen 2014^5^	Low risk	Low risk	Low risk	Low risk	Unclear	Unclear
Parker 2002^20^; Parker 2010^39^	Low risk	Low risk	Low risk	Low risk	Unclear	Unclear
Parker 2010^40^	Low risk	Low risk	Low risk	Low risk	Unclear	Unclear
Puolakka 2001^38^	Unclear	Low risk	High risk	Low risk	Unclear	Unclear
Raia 2003^37^	Low risk	Unclear	Low risk	Low risk	Unclear	Unclear
Ravikumar 2000^36^	Unclear	Unclear	Unclear	Low risk	Unclear	Unclear
Rödén 2003^35^	Unclear	Low risk	High risk	Low risk	Unclear	Unclear
Santini 2005^34^	Unclear	Unclear	Unclear	High risk	Unclear	Unclear
Sikorski 1981^33^	Low risk	Unclear	Unclear	Low risk	Unclear	Unclear
Skinner 1989^32^	Unclear	Unclear	High risk	Low risk	Unclear	Unclear
Somashekar 2013^6^	Unclear	Unclear	Unclear	High risk	Unclear	Unclear
Stoffel 2013^31^	Low risk	Unclear	Low risk	Low risk	Unclear	Unclear
Taylor 2012^30^	Low risk	Low risk	Low risk	Low risk	Unclear	Unclear
van den Bekerom 2010^28^	Low risk	Unclear	High risk	Low risk	Unclear	Unclear
van Vugt 1993^26^	Unclear	Unclear	High risk	High risk	Unclear	Unclear

### Evidence Network

**Figure 2:**
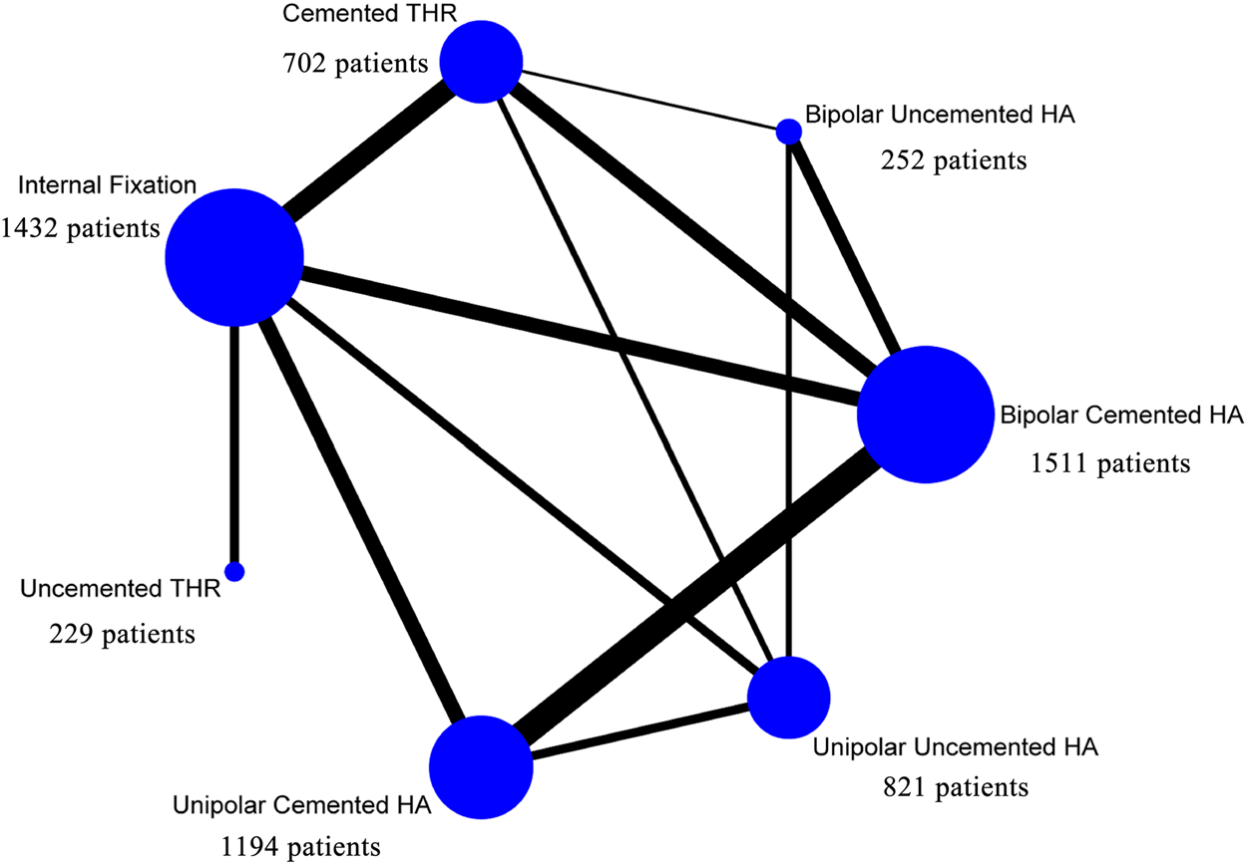
Direct comparisons in the network model.

The various procedures and number of studies and patients per direct comparison included in our network-analysis are shown in Fig. 2. The thickness of lines represents the number of studies; and the blue spots, the number of patients.

## Primary End Point

### Reoperation

**Figure 3:**
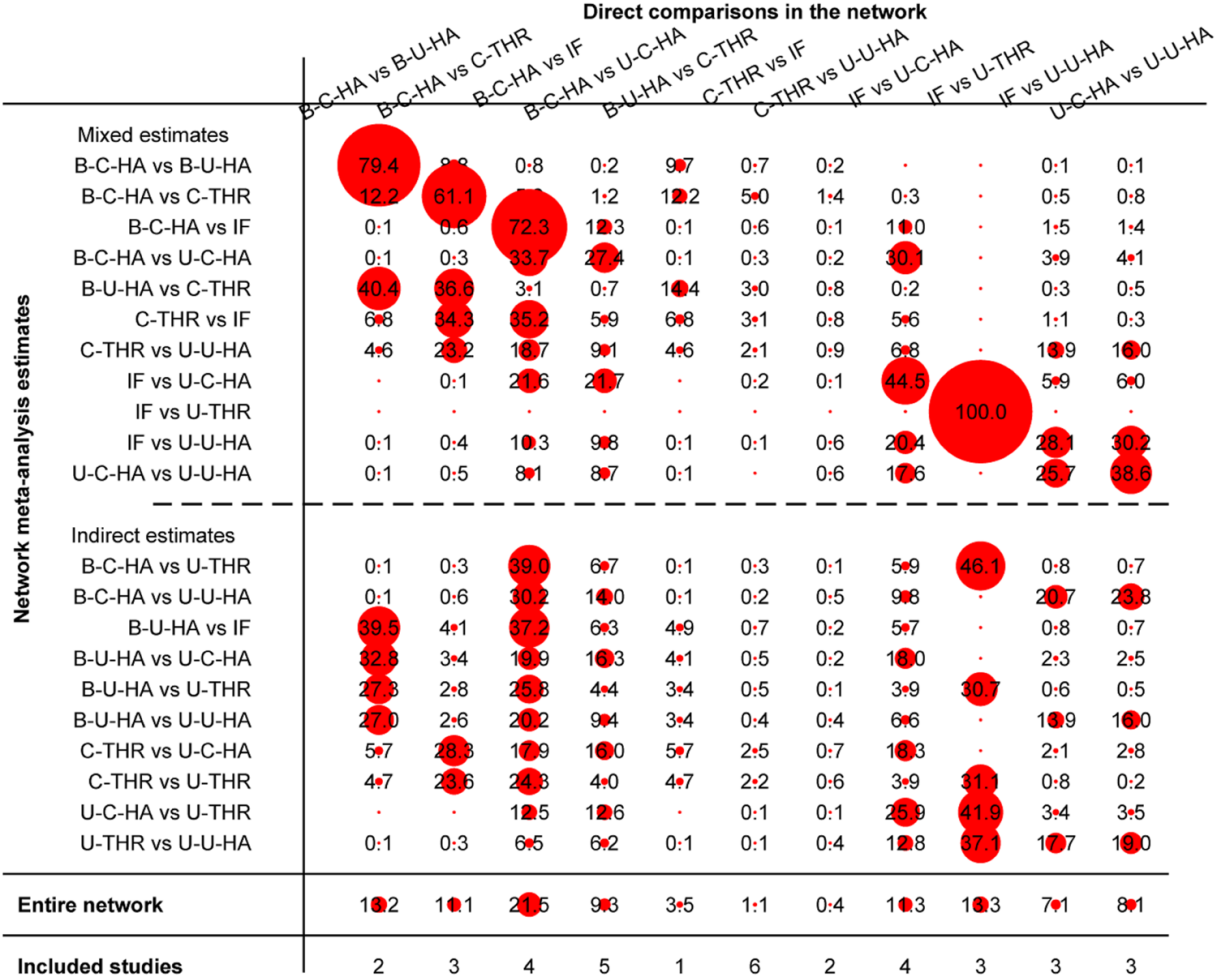
Contribution of the direct estimates effect of each pair in the entire network for reoperation (IF, internal fixation; unipolar-cemented-HA, U-C-HA; unipolar-uncemented-HA, U-U-HA; bipolar-cemented-HA, B-C-HA; bipolar-uncemented-HA, B-U-HA; uncemented-THR, U-THR; cemented-THR, C-THR).

The contribution of the direct estimates effect of each pair in the entire network is shown in Fig. 3. Mixed estimates included direct and indirect estimates; the indirect estimate was constructed via intermediary. For example, in the mixed estimates of reoperation, the direct comparison result of bipolar cemented HA versus bipolar uncemented HA provided 79.4% weights for the mixed estimates, and this pair contributed a proportion of 39.5% to the result of bipolar cemented HA versus IF, a proportion of 32.8% to the result of bipolar cemented HA versus unipolar cemented HA, a proportion of 27.3% to the result of bipolar uncemented HA versus uncemented THR, and so on. In the mixed estimates reoperation of bipolar cemented HA versus cemented THR, the proportion from direct comparison was 61.1%. This pair contributed 28.3% weights to the result of cemented THR versus unipolar cemented HA and 23.6% to the result of cemented THR versus uncemented THR. In the network, other pairs were similar to the above comparisons.

**Figure 4:**
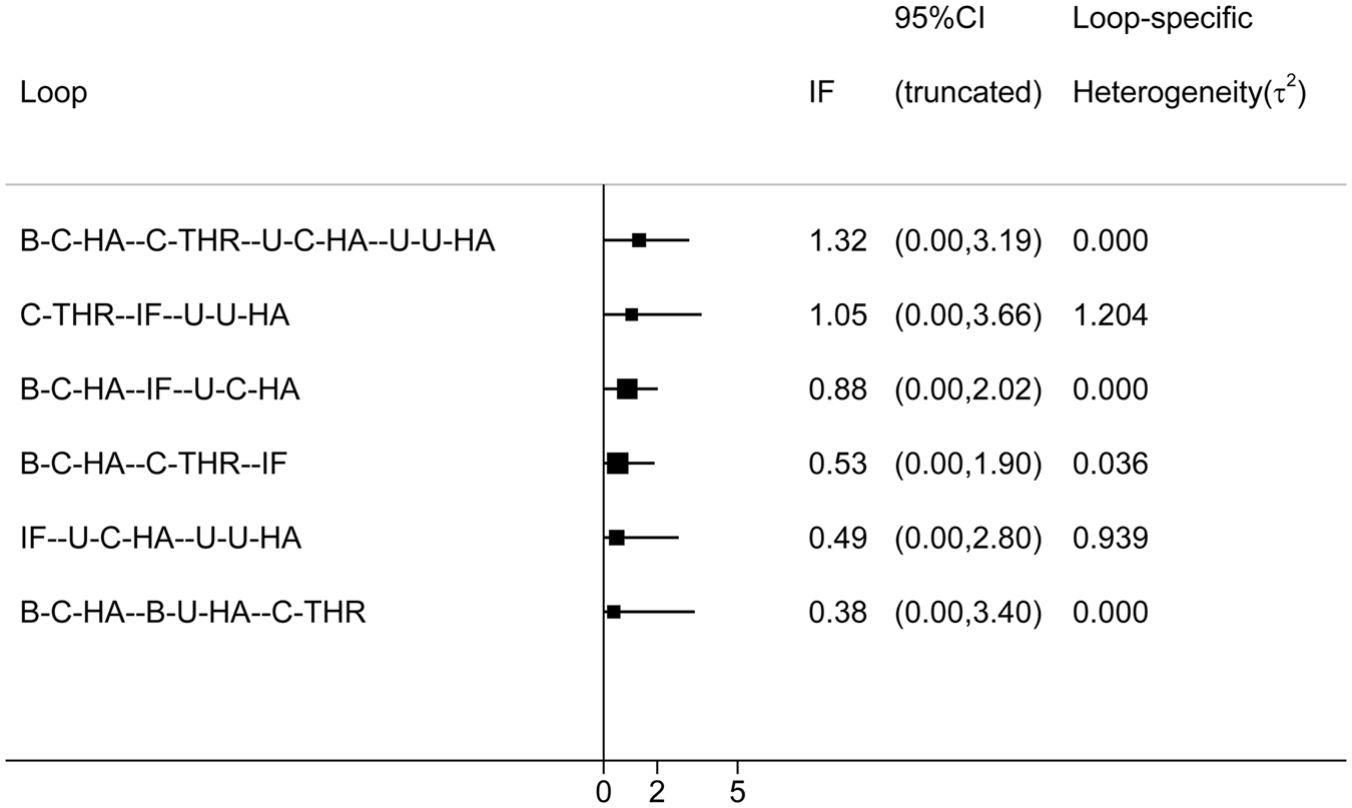
Inconsistency in the closed loops of reoperation. (IF, internal fixation; unipolar-cemented-HA, U-C-HA; unipolar-uncemented-HA, U-U-HA; bipolar-cemented-HA, B-C-HA; bipolar-uncemented-HA, B-U-HA; uncemented-THR, U-THR; cemented-THR, C-THR).

Inconsistency in closed loops was assessed using inconsistency factors (Fig. 4). Any 3 treatments forming a direct triangular connection were assessed for inconsistency. If the lower limit of the 95% confidence interval in any 1 of the closed loops did not reach 0, this insinuates that a statistical difference may exist in the inconsistency hypothetical test. No loop indicated the possibility of inconsistency (Fig. 4).

Table 3 displays the effect estimates of reoperation (lower left, italic). Overall, the results showed that the reoperation incidence was higher in the IF group than in the other groups (compared to unipolar cemented HA [OR = 13.39; 95% CrI 6.21 to 26.06], unipolar uncemented HA [OR = 3.80; 95% CrI 1.72 to 7.43], bipolar cemented HA [OR = 8.10; 95% CrI 4.20 to 14.16], bipolar uncemented HA [OR = 6.87; 95% CrI 1.16 to 23.30], uncemented THR [OR = 9.93; 95% CrI 3.13 to 25.23], and cemented THR [OR = 11.45; 95% CrI 5.48 to 21.88]). The reoperation incidence was lower in the unipolar cemented HA group than in the unipolar uncemented HA group (OR = 0.31; 95% CrI 0.12 to 0.66). In the remainder of comparisons, the results did not indicate any significant differences. Based on the above outcomes, we drew the rank of these 7 procedures under the reoperation incidence using surface under the cumulative ranking curve (SUCRA). The rank of potential reoperation from low incidence to high incidence was as follows: 1 for unipolar cemented HA; 2, cemented THR; 3, uncemented THR; 4, bipolar cemented HA; 5, bipolar uncemented HA; 6, unipolar uncemented HA; and 7, IF.

**Table 3:** Main findings of reoperation and mortality.

IF	**1.01(0.78, 1.29)**	**1.07(0.82, 1.37)**	**0.91(0.71, 1.16)**	**0.93(0.40, 1.83)**	**1.07(0.69, 1.60)**	**1.08(0.79, 1.43)**
*13.39(6.21, 26.06)*	unipolar-cemented-HA	**1.07(0.81, 1.38)**	**0.91(0.69, 1.19)**	**0.93(0.40, 1.86)**	**0.84(0.64, 1.72)**	**1.08(0.76, 1.50)**
*3.80(1.72, 7.43)*	*0.31(0.12, 0.66)*	unipolar-uncemented-HA	**0.87(0.63, 1.17)**	**0.88(0.37, 1.78)**	**1.02(0.60, 1.63)**	**1.02(0.71, 1.41)**
*8.10(4.20, 14.16)*	*0.66(0.29, 1.27)*	*2.41(0.90, 5.20)*	bipolar-cemented-HA	**1.02(0.46, 1.95)**	**1.20(0.71, 1.89)**	**1.19(0.87, 1.58)**
*6.87(1.16, 23.30)*	*0.56(0.09, 1.92)*	*2.04(0.29, 7.43)*	*0.85(0.17, 2.69)*	bipolar-uncemented-HA	**1.35(0.51, 2.91)**	**1.34(0.57, 2.69)**
*9.93(3.13, 25.23)*	*0.84(0.20, 2.47)*	*3.00(0.70, 8.94)*	*1.35(0.34, 3.86)*	*2.60(0.27, 10.51)*	uncemented-THR	**1.05(0.61, 1.70)**
*11.45(5.48, 21.88)*	*0.96(0.34, 2.17)*	*3.37(1.25, 7.42)*	*1.51(0.64, 3.13)*	*2.86(0.44, 9.67)*	*1.53(0.35, 4.31)*	cemented-THR

Comparisons of reoperation are on the lower left (italic), with mortality on the top right (bold).

### Mortality

**Figure 5:**
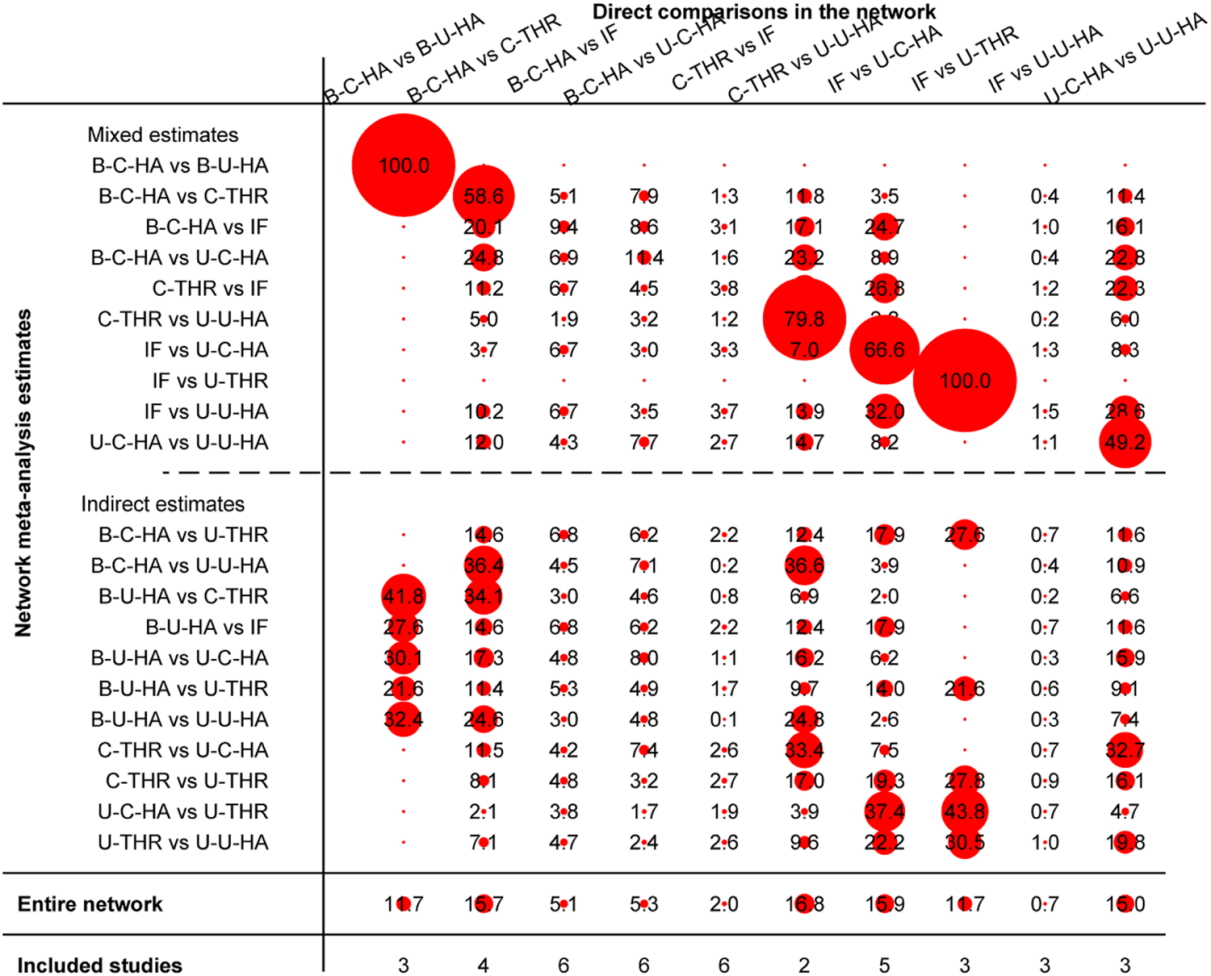
Contribution of the direct estimates effect of each pair in the entire network for mortality (IF, internal fixation; unipolar-cemented-HA, U-C-HA; unipolar-uncemented-HA, U-U-HA; bipolar-cemented-HA, B-C-HA; bipolar-uncemented-HA, B-U-HA; uncemented-THR, U-THR; cemented-THR, C-THR).

**Figure 6:**
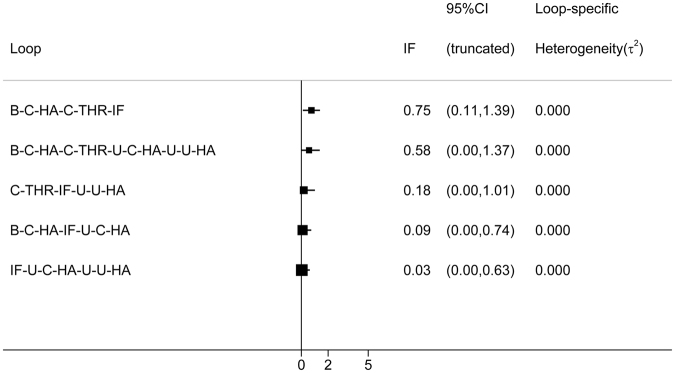
The inconsistency in closed loops of mortality (IF, internal fixation; unipolar-cemented-HA, U-C-HA; unipolar-uncemented-HA, U-U-HA; bipolar-cemented-HA, B-C-HA; bipolar-uncemented-HA, B-U-HA; uncemented-THR, U-THR; cemented-THR, C-THR).

The contribution each pair in the entire network is shown in Fig. 5. The inconsistency in closed loops was assessed using inconsistency factors (Fig. 6). Notably, the bipolar cemented HA-cemented THR-IF loop indicated the possibility of inconsistency, as the 95% confidence interval was 0.11 to 1.39, *P* = 0.021. Table 3 also displays the effect estimates of mortality (top right, bold). Mortality during the follow-up did not differ significantly. Overall, the results showed that the mortality incidence in these 7 groups was similar. Thus, no drawing or ranking of order under mortality was necessary.

### Infection

**Figure 7:**
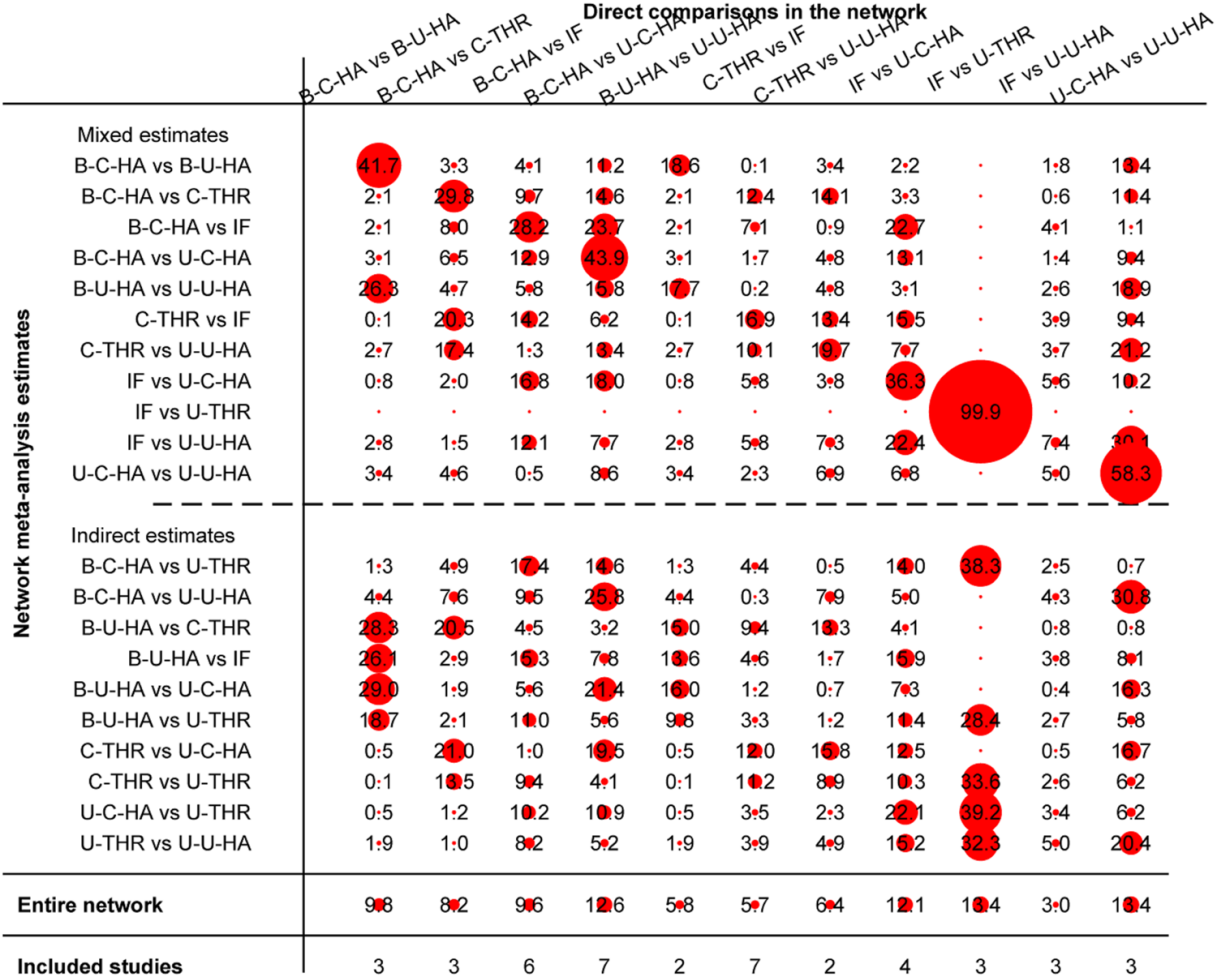
Contribution of the direct estimates effect of each pair in the entire network for infection (IF, internal fixation; unipolar-cemented-HA, U-C-HA; unipolar-uncemented-HA, U-U-HA; bipolar-cemented-HA, B-C-HA; bipolar-uncemented-HA, B-U-HA; uncemented-THR, U-THR; cemented-THR, C-THR).

**Figure 8:**
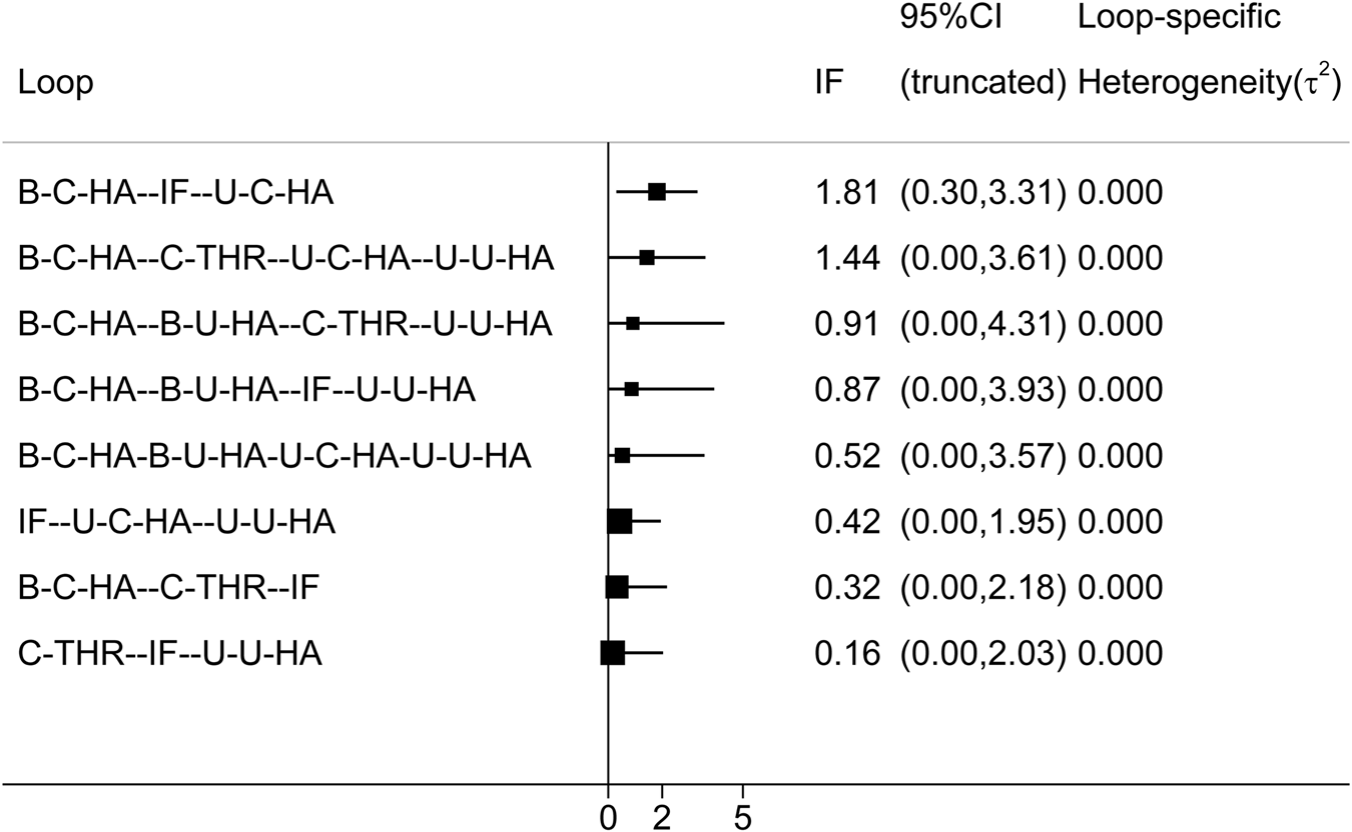
Inconsistency in closed loops of infection (IF, internal fixation; unipolar-cemented-HA, U-C-HA; unipolar-uncemented-HA, U-U-HA; bipolar-cemented-HA, B-C-HA; bipolar-uncemented-HA, B-U-HA; uncemented-THR, U-THR; cemented-THR, C-THR).

The contribution of each pair in the entire network is shown in Fig. 7. The inconsistency in closed loops was assessed using inconsistency factors (Fig. 8). The bipolar cemented HA-IF-unipolar cemented HA loop indicated potential inconsistency (*P* = 0.019). Table 4 displays the effect estimates for infection and dislocation. Regarding infection, a few significant differences were observed. Overall, the results showed that the infection incidence in IF group was lower than that in the unipolar uncemented HA group (OR = 0.49; 95% CrI 0.20 to 0.99). In the bipolar uncemented HA group, the infection incidence was lower than that in the IF (OR = 39.48; 95% CrI 1.00 to 203.4), unipolar cemented HA (OR = 70.60; 95% CrI 1.88 to 391.30), unipolar uncemented HA (OR = 95.06; 95% CrI 2.16 to 443.90), bipolar cemented HA (OR = 70.57; 95% CrI 1.94 to 362.20), uncemented THR (OR = 0.07; 95% CrI 0.00 to 0.44), and cemented THR groups (OR = 0.18; 95% CrI 0.00 to 0.86). In the remainder of comparisons, the results did not indicate significant difference. Based on the above outcomes, we drew the rank of these 7 procedures by “SUCRA”. The rank of potential infection, from low to high incidence, was as follows: 1 for bipolar uncemented HA; 2, IF; 3, cemented THR; 4, bipolar cemented HA; 5, unipolar cemented HA; 6, unipolar uncemented HA; and 7, uncemented THR.

**Table 4:** Main findings of infection and dislocation.

IF	0.23(0.04, 0.68)	0.20(0.05, 0.53)	0.46(0.13, 1.13)	0.59(0.08, 2.25)	0.003(0.00, 0.03)	0.08(0.02, 0.19)
*0.55(0.24, 1.08)*	unipolar-cemented-HA	**1.20(0.23, 3.76)**	**2.53(0.81, 6.20)**	**3.40(0.41, 13.39)**	**0.02(0.00, 0.15)**	**0.50(0.10, 1.52)**
*0.49(0.20, 0.99)*	*0.97(0.40, 1.96)*	unipolar-uncemented-HA	**2.92(0.68, 8.35)**	**3.60(0.48, 13.70)**	**0.02(0.00, 0.18)**	**0.50 (0.14, 1.21)**
*0.58(0.25, 1.11)*	*1.15(0.50, 2.29)*	*1.33(0.49, 3.06)*	bipolar-cemented-HA	**1.37(0.24, 4.60)**	**0.01(0.00, 0.08)**	**0.21(0.06, 0.51)**
*39.48(1.00, 203.4)*	*70.60(1.88, 391.30)*	*95.06(2.16, 443.90)*	*70.57(1.94, 362.20)*	bipolar-uncemented-HA	**0.01(0.00, 0.07)**	**0.24(0.03, 0.83)**
*0.30(0.02, 1.07)*	*0.63(0.04, 2.49)*	*0.71(0.04, 2.87)*	*0.60(0.04, 2.36)*	*0.07(0.00, 0.44)*	uncemented-THR	**6.44E+32(2.52,1.07E+30)**
*0.80(0.30, 1.73)*	*1.64(0.49, 3.90)*	*1.83(0.59, 4.36)*	*1.52(0.49, 3.62)*	*0.18(0.00, 0.86)*	*8.04(0.55, 37.74)*	cemented-THR

Comparisons of infection are on the lower left (italic), with dislocation on the top right (bold).

### Dislocation

**Figure 9:**
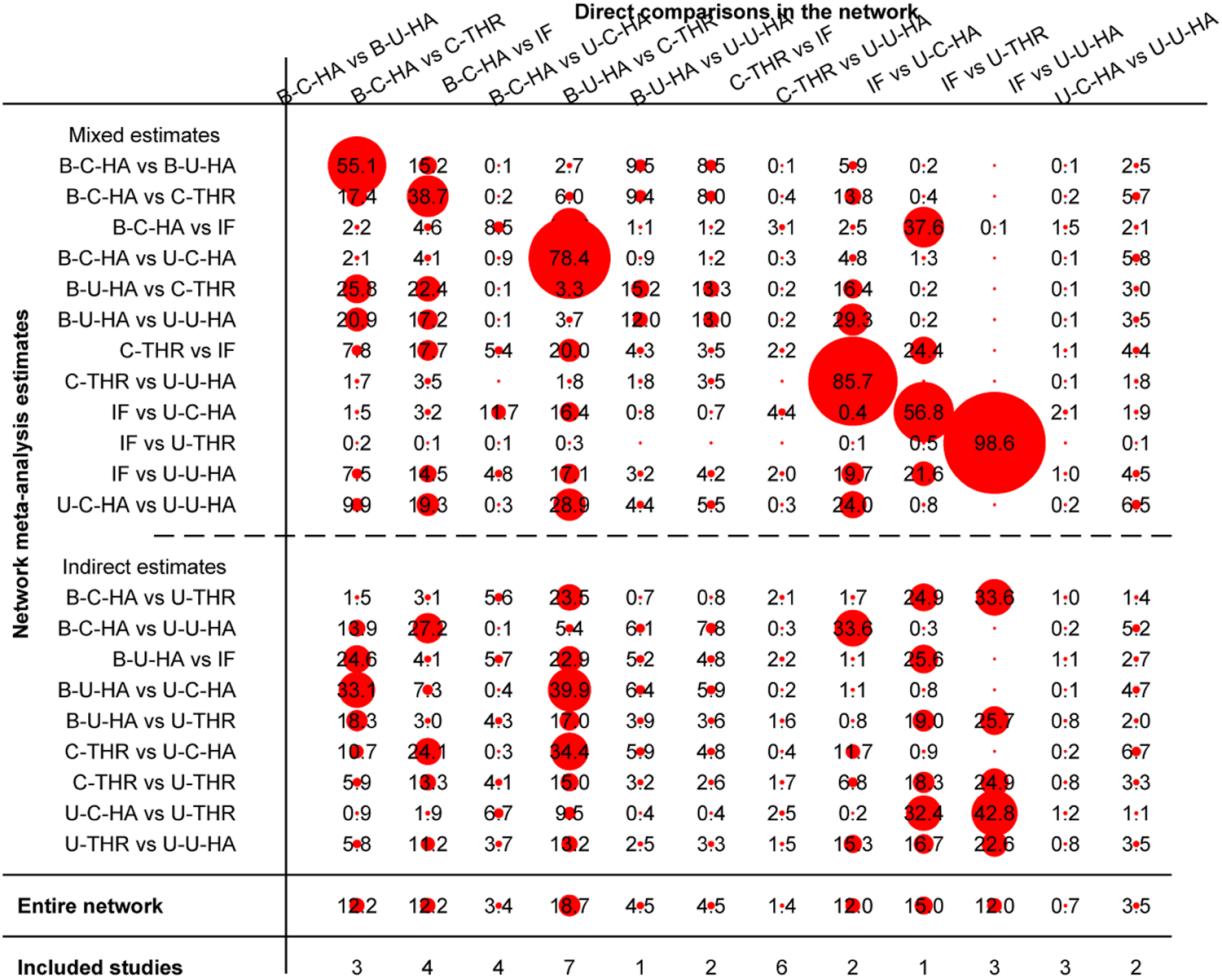
Contribution of the direct estimates effect of each pair in the entire network for dislocation (IF, internal fixation; unipolar-cemented-HA, U-C-HA; unipolar-uncemented-HA, U-U-HA; bipolar-cemented-HA, B-C-HA; bipolar-uncemented-HA, B-U-HA; uncemented-THR, U-THR; cemented-THR, C-THR).

**Figure 10:**
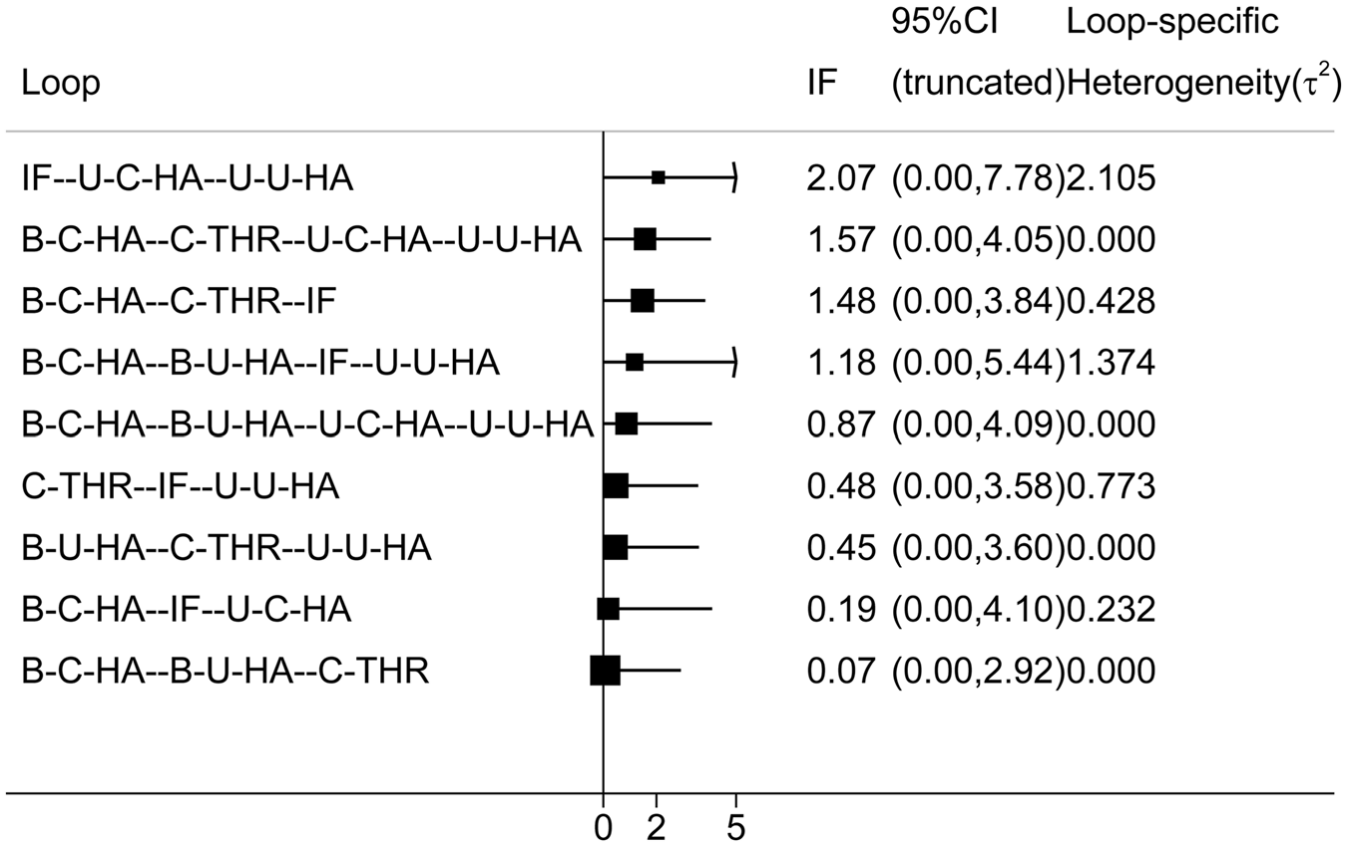
Inconsistency in closed loops of dislocation (IF, internal fixation; unipolar-cemented-HA, U-C-HA; unipolar-uncemented-HA, U-U-HA; bipolar-cemented-HA, B-C-HA; bipolar-uncemented-HA, B-U-HA; uncemented-THR, U-THR; cemented-THR, C-THR).

The contribution of each pair in the entire network is shown in Fig. 9. The inconsistency in closed loops was assessed using inconsistency factors (Fig. 10). Table 4 also displays the effect estimates of dislocation. For dislocation during follow-up, a few significant differences were observed. Overall, the results showed that the dislocation rate in the IF group was lower than that in the other groups (compared to unipolar cemented HA [OR = 0.23; 95% CrI 0.04 to 0.68] and unipolar uncemented HA [OR = 0.20; 95% CrI 0.05 to 0.53]). The dislocation incidence was higher in the uncemented THR group than in the IF (OR = 0.003; 95% CrI 0.00 to 0.03), unipolar cemented HA (OR = 0.02; 95% CrI 0.00 to 0.15), unipolar uncemented HA (OR = 0.02; 95% CrI 0.00 to 0.18), bipolar cemented HA (OR = 0.01; 95% CrI 0.00 to 0.08), bipolar uncemented HA (OR = 0.01; 95% CrI 0.00 to 0.07), and cemented THR groups (OR = 6.44E + 32; 95% CrI 2.52 to 1.07E + 30). The dislocation incidence was higher in the cemented THR group than in the IF (OR = 0.08; 95% CrI 0.02 to 0.19), bipolar cemented HA (OR = 0.21; 95% CrI 0.06 to 0.51), and bipolar uncemented HA groups (OR = 0.24; 95% CrI 0.03 to 0.83). In the remainder of the comparisons, the results did not indicate significant difference. Based on the above outcomes, we drew the rank of these 7 procedures regarding infection incidence by “SUCRA”. The rank of potential dislocation, from low incidence to high incidence, was as follows: 1 for IF; 2, bipolar uncemented HA; 3, bipolar cemented HA; 4, unipolar cemented HA; 5, unipolar uncemented HA; 6, cemented THR; and 7, uncemented THR.

## Discussion

Traditionally, the selection of the appropriate treatment for patients should depend on the age, location of fracture, orientation, comminution, type, stability, and requirements of postoperative functional recovery and so on. In this study, we used Bayesian network meta-analysis to compare efficacy and complications between 7 procedures.

Reoperation is an important end point of efficacy. The results demonstrate that patients in the IF group had the highest incidence of arthroplasty. Simultaneously, the unipolar cemented HA group exhibited a lower incidence than did the unipolar uncemented HA group and may provide the lowest reoperation incidence in all of managements, the next order is cemented-THR. In addition, procedures with bone cement tend to have lower reoperation incidence than those without cement: unipolar cemented HA < unipolar uncemented HA, bipolar cemented HA < bipolar uncemented HA, and cemented THR < uncemented THR. This suggests a role for cement in fixing stems and reducing the requirement for reoperation, which may be protective for patients. In additional, Inngul *et al*. reported that bipolar HA could display a later onset of acetabular erosion compared to unipolar HA^10,56^. Thus, the reoperation incidence in the bipolar HA group is expected to be at least the equivalent to unipolar HA. Two meta-analyses comparing unipolar and biopolar HA support this conjecture^57,58^. But in our analysis, unipolar cemented HA exhibited lower reoperation incidence than other managements, and notably lower than did bipolar HA. We think that cementing has such a large influence on unipolar HA that unipolar cemented HA exhibits a lower reoperation incidence than do other managements. Typically, the main reason for reoperation in IF is loss of fixation, whilst in arthroplasty, it is the revision typically required following acetabular erosion, dislocation, periprosthetic fracture, pain, and loosening^10,21,59^.

The rank of dislocation incidence in our meta-analysis shows another tendency: IF < bipolar HA < unipolar HA < THR. The IF group exhibited the lowest dislocation rate because of a tendency to fail in fixation rather than dislocation. THR had a higher dislocation incidence because the existence of an acetabular cup may destroy bone mass. Uncemented THR contributed the highest dislocation incidence, when compared to other groups, in this meta-analysis. Further, low dislocation incidence in bipolar HA corresponds with the theory that bipolar HA may reduce the amount of acetabular erosion, compared to unipolar HA^60^.

Mortality constitutes another important clinical factor, and we found that there was no difference between interventions, which indicates that the type of operation does not obviously influence mortality. Complications illustrate that the infection incidence in bipolar uncemented HA is the lowest of all managements, a significant difference compared to other groups. Unfortunately, no immediate explanation is apparent for these findings.

Our meta-analysis has several potential limitations that should be considered. First, Bayesian network meta-analysis is an emerging and encouraging research method, which is not very perfect and convincing, but we could not completely refuse the evidence from it because it provided direct and indirect estimates, and the possible order of managements. In our meta-analysis, the 95% CrI varied greatly, and we suggest that this may be due to differences in RCTs. Indicators were analyzed and discussed as the results of ranking, which provides a tendency, and the contribution from direct comparison is only a little part of all. Therefore, systematic bias is possible in this study. Second, confounding factors involving surgical approach and variation from IF may contribute to differences in the findings. A direct anterior (Smith-Peterson), anterolateral (Watson-Jones), lateral (Hardinge), posterior (Moore), or posterolateral approach could be used to perform hip arthroplasty^38,61–64^. Although meta-analyses have reported similar outcomes and complication incidences between operative approaches so far^65,66^, a higher dislocation incidence may exist when using the posterior approach^67^, contributing heterogeneity to the overall outcome. In addition, IF is divided into closed and open reduction depending on whether cancellous lag screws or dynamic hip screws are used, which is another source of heterogeneity. Finally, according to the methodological quality items in Cochrane Reviewers’ Handbook 5.1.0^24^, different bias might be introduced in the study, especially selective reporting bias, because all of studies were unclear. We should be cautious to the conclusion.

In conclusion, our network meta-analysis rank the orders of 7 procedures in reoperation, mortality, dislocation and infection, which indicates that IF may provide the highest reoperation incidence, unipolar cemented HA may provide the lowest reoperation incidence; uncemented THR contributes the highest dislocation incidence; and bipolar uncemented HA provides the lowest infection incidence. No differences in mortality were observed among the treatments. This conclusion is indirect; higher-quality direct comparisons are required.

## Electronic supplementary material

Supplementary Table S1
