# Supplementary material for: Interventions for treating displaced intracapsular femoral neck fractures in the elderly: a Bayesian network meta-analysis of randomized controlled trials
Source: Sci Rep. 2017 Oct 12;7:13103. doi: 10.1038/s41598-017-13377-1 (PMC5638843; doi:10.1038/s41598-017-13377-1)
Supplement: Supplementary file 1 — Supplementary Table S1 [file 41598_2017_13377_MOESM1_ESM.pdf]

Interventions for treating displaced intracapsular femoral neck fractures in the elderly: a Bayesian network meta-analysis of randomized controlled trials

Bin-Fei Zhang, Peng-Fei Wang, Hai Huang, Yu-Xuan Cong, Hu Wang, Yan Zhuang

Supplementary Table S1. The search strategy for Medline (Pubmed):

#26	Search #24 AND #25
#25	Search #5 OR #8 OR #9 OR #10 OR #11 OR #12 OR #14 OR #15 OR #18 OR #19 OR #20
#24	Search #1 OR #3 OR #4
#20	Search randomly allocation*
#19	Search random allocation*
#18	Search "Random Allocation"[Mesh]
#15	Search double blind*
#14	Search "Double-Blind Method"[Mesh]
#12	Search single blind*
#11	Search "Single-Blind Method"[Mesh]
#10	Search "Randomized Controlled Trial" [Publication Type] OR "Randomized Controlled Trials as Topic"[Mesh] OR "Controlled Clinical Trial" [Publication Type]
#9	Search randomized controlled trial*
#8	Search "Randomized Controlled Trial"
#5	Search randomized controlled trial
#4	Search "Femoral Neck Fractures"
#3	Search "Femoral Neck Fractures"[Mesh]
#1	Search Femoral Neck Fractures
